# Supplementary material for: Latiné immigrant heterogeneity: Striking health differences among Cuban refugee/migration waves to the United States
Source: medRxiv. 2025 Sep 19:2024.04.17.24305856. Originally published 2024 Apr 19. Preprint. [Version 2] doi: 10.1101/2024.04.17.24305856 (PMC11065033; doi:10.1101/2024.04.17.24305856)
Supplement: Supplement 1 [file media-1.pdf]

Variables	Early Cuban Exiles	Freedom Flights	Pre- and Post-Mariel Boatlift	Special Period
N	87	105	106	168
Age	62.49 ± 12.53	57.22 ± 13.88	51.72 ± 16.60	42.45 ± 15.60
Females (N)	48	65	51	83
Males (N)	39	40	55	85
Northeast (N)	3	14	6	5
South (N)	83	90	100	163
West (N)	1	1	0	0

Supplementary Figure 1. Descriptive statistics for Cuban subsample of NLAAS. Here, we report general descriptive statistics for the Cuban subsample of the NLAAS study. Average age and standard deviation was generated while incoroporating sampling weights from the NLAAS dataset (see Methods). Note: The individuals included in these analyses are those that met the cutoffs for the migration waves of interest for this study, therefore not all individuals that identify as Cuban in NLAAS are present in these statistics.

Health Measures	Early Cuban Exiles	Freedom Flights	Pre- and Post-Mariel Boatlift	Special Period
N	87	105	106	168
CARESCORE	1.85 ± 10.08	1.07 ± 6.43	3.47 ± 12.68	0.82 ± 5.76
COGSCORE	1.08 ± 5.86	3.17 ± 12.10	4.67 ± 13.47	1.71 ± 8.58
MOVESCORE	5.61 ± 17.63	4.60 ± 14.73	8.52 ± 20.51	3.63 ± 14.26
OUTROLESCORE	7.96 ± 22.54	10.35 ± 26.88	21.74 ± 39.42	9.45 ± 25.42
SOCIALSCORE	0.64 ± 3.39	1.39 ± 7.05	3.18 ± 12.61	0.81 ± 5.14
DISABILITYSCORE	17.14 ± 45.83	20.58 ± 53.38	41.59 ± 81.76	16.41 ± 44.13
Disabled (N)	23	38	39	37
Not Disabled (N)	64	67	67	131

Supplementary Figure 2. Health variable summary statistics for Cuban subsample of NLAAS. This table reports the average, standard deviation, and N (where applicable) for the health measures of interest in this study for the Cuban cohort. These results were generated while incoroporating sampling weights from the NLAAS dataset (see Methods). Note: The individuals included in these analyses are those that met the cutoffs for the migration waves of interest for this study, therefore not all individuals that identify as Cuban in NLAAS are present in these statistics. The composite disability score (DISABILITYSCORE) was calculated by adding together all five WHODAS scores for each individual. Individuals were categorized as disabled if they had a composite disability score greater than zero.

Health Measures	Early Cuban Exiles	Freedom Flights	Pre- and Post-Mariel Boatlift	Special Period
Medical Discrimination	1.00 ± 0.00	1.34 ± 0.83	1.49 ± 1.12	1.14 ± 0.47
Everyday Discrimination	50.98 ± 5.18	50.69 ± 4.71	50.37 ± 5.74	50.50 ± 5.05

Supplementary Figure 3. Discrimination scores summary statistics for the Cuban subsample of NLAAS. This table reports the averages and standard deviations for Medical Discrimination and Everyday Discrimination scores of the Cuban cohort. Averages and standard deviations were generated while incorporating sampling weights from the NLAAS dataset (see Methods). Note: The individuals included in these analyses are those that met the cutoffs for the migration waves of interest for this study, therefore not all individuals that identify as Cuban in NLAAS are present in these statistics.

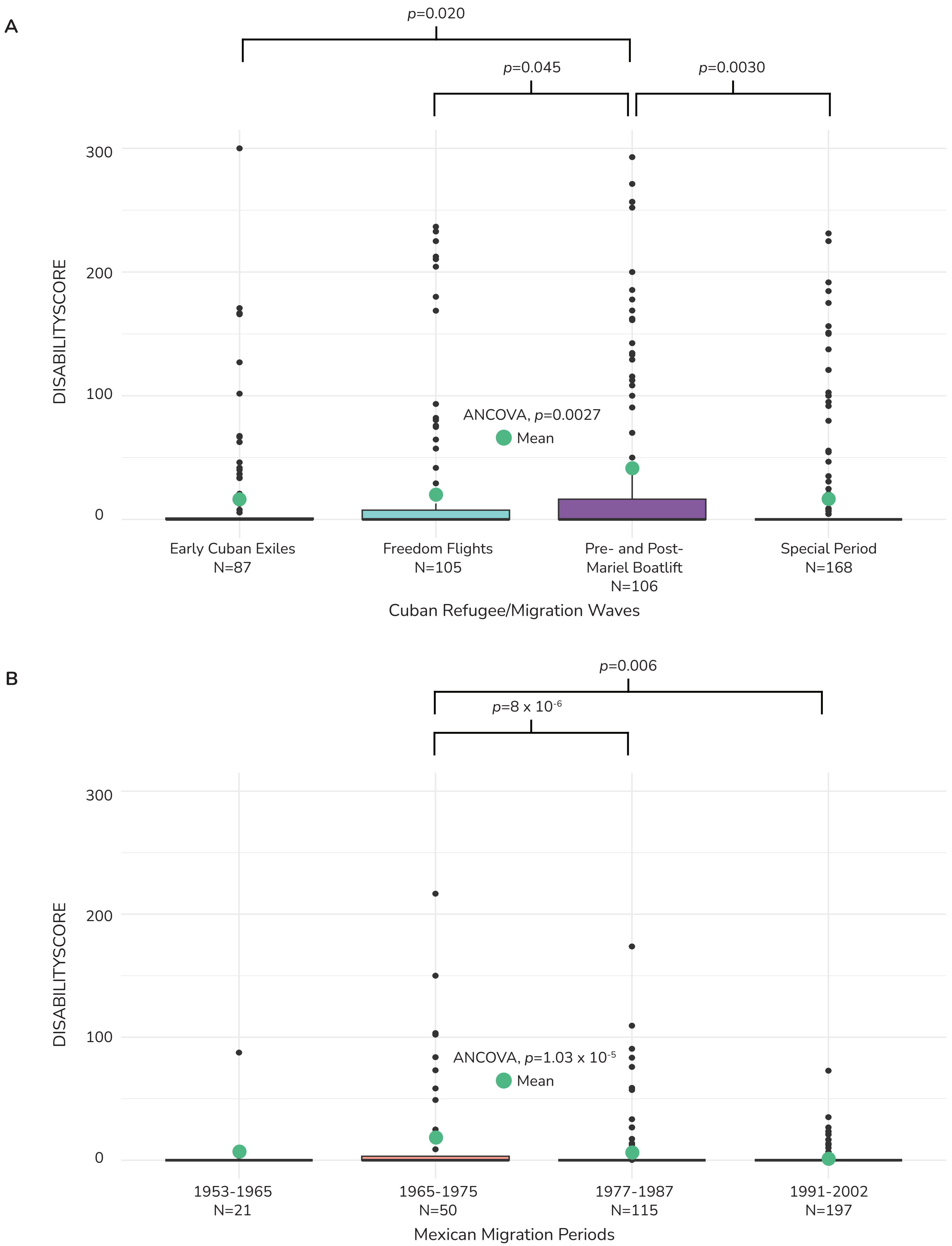

Supplementary Figure 4. Composite disability scores for Cubans and Mexicans in NLAAS. (**A**) Boxplot of distribution of values and average for the composite disability score (DISABILITYSCORE) across Cuban refugee/migration waves. (**B**) Boxplot of distribution of values and average for the composite disability score (DISABILITYSCORE) across Mexican migration periods that correspond to Cuban refugee/migration waves.

Health Measures	1953-1964	1965-1975	1977-1987	1991-2002
Medical Discrimination	0.00 ± 0.00	1.63 ± 1.15	1.17 ± 0.59	1.49 ± 0.58
Everyday Discrimination	50.39 ± 7.87	55.22 ± 4.18	51.38 ± 8.57	50.98 ± 7.93

Supplementary Figure 5. Discrimination scores summary statistics for the Mexican subsample of NLAAS. This table reports the averages and standard deviations for Medical Discrimination and Everyday Discrimination scores of the Mexican cohort. Averages and standard deviations were generated while incorporating sampling weights from the NLAAS dataset (see Methods).

**Health Score**	CARESCORE	COGSCORE	MOVESCORE	OUTROLE-SCORE	SOCIALSCORE	DISABILITY-SCORE
CARESCORE						
COGSCORE	r = 0.32 p < 2.2e-16					
MOVESCORE	r = 0.56 p < 2.2e-16	r = 0.39 p < 2.2e-16				
OUTROLESCORE	r = 0.40 p < 2.2e-16	r = 0.45 p < 2.2e-16	r = 0.59 p < 2.2e-16			
SOCIALSCORE	r = 0.43 p < 2.2e-16	r = 0.54 p < 2.2e-16	r = 0.38 p < 2.2e-16	r = 0.37 p < 2.2e-16		
DISABILITYSCORE	r = 0.63 p < 2.2e-16	r = 0.62 p < 2.2e-16	r = 0.81 p < 2.2e-16	r = 0.91 p < 2.2e-16	r = 0.58 p < 2.2e-16	

Supplementary Table 1. Correlation coefficients for NLAAS health measures analyzed in this study. Here, we report correlation coefficents and p-values for each Pearson’s Correlation Test conducted between health measures used within this study. All health measures were significantly positively correlated with one another.

Question	Variable	Range	Scale
Frequency treated with less courtesy than others	DS1A	1-6	1: Almost everyday<br>2: At least once a week<br>3: A few times a month<br>4: A few times a year<br>5: Less than once a year<br>6: Never
Frequency treated with less respect than others	DS1B		
Frequency received poorer restaurant service than others	DS1C		
Frequency people act like you are not smart	DS1D		
Frequency people act afraid of you	DS1E		
Frequency people act like you are dishonest	DS1F		
Frequency people act better than you	DS1G		
Frequency called names/insulted	DS1H		
Frequency threatened/harrassed	DS1I		

Supplementary Table 2. Everyday Discrimination Score (EDS) survey questions. Survey questions used to generate the Everyday Discrimination Score (EDS) used as a measure of overall discrimination as part of this study. Participants were the frequency with which they experience discrimination on an everyday basis, with six potential responses: almost everyday, at least once a week, a few times a month, a few times a year, less than once a year, or never. These responses were used to create a numerical value, with a lower value indicating more discrimination and a higher value indicating less discrimination. The scores for survey items DS1A-DS1I were added together to a create a compostion Everyday Discrimination Score, with a range of 9-54.

Dependent Variable (Presence/Absence)	Description	Independent Variable	Control Variables	Odds Ratio	p-value
CARESCORE	An individual's ability to tend to their own personal hygiene	Medical Discrimination (FD21)	Age, Sex, Region	1.89	0.050
COGSCORE	An individual's ability to understand, interact, and cognitively process the world around them	Medical Discrimination (FD21)	Age, Sex, Region	1.85	0.041
MOVESCORE	An individual's ability to move and get around within the home and outside in the wider environment	Medical Discrimination (FD21)	Age, Sex, Region	N/A	0.85
OUTROLESCORE	an individual's ability to complete daily life activities such as work, household activities, and caring for family	Medical Discrimination (FD21)	Age, Sex, Region	N/A	0.33
SOCIALSCORE	An individual's capacity for interpersonal inter-actions	Medical Discrimination (FD21)	Age, Sex, Region	3.87	0.023
DISABILITYSCORE	Composite disability score	Medical Discrimination (FD21)	Age, Sex, Region	N/A	0.94
CARESCORE	An individual's ability to tend to their own personal hygiene	Everyday Discrimination (EDS)	Age, Sex, Region	N/A	0.064
COGSCORE	An individual's ability to understand, interact, and cognitively process the world around them	Everyday Discrimination (EDS)	Age, Sex, Region	N/A	0.069
MOVESCORE	An individual's ability to move and get around within the home and outside in the wider environment	Everyday Discrimination (EDS)	Age, Sex, Region	N/A	0.25
OUTROLESCORE	An individual's ability to complete daily life activities such as work, household activities, and caring for family	Everyday Discrimination (EDS)	Age, Sex, Region	N/A	0.33
SOCIALSCORE	An individual's capacity for interpersonal inter-actions	Everyday Discrimination (EDS)	Age, Sex, Region	N/A	0.073
DISABILITYSCORE	Composite disability score	Everyday Discrimination (EDS)	Age, Sex, Region	N/A	0.091

Supplementary Table 3. Logistic regression results for discrimination scores. Here we show the results of the logistic regression models for each health measure converted to a binary variable. If a score across any domain was more than zero, those individuals were coded with the presence of a disability. We test if medical discrimination and everyday discrimination scores are associated with the presence of diability, while controlling for age, sex, and region across each model. All models include NLAAS sampling weights.
