# Supplementary material for: Latiné immigrant heterogeneity: Striking health differences among Cuban refugee/migration waves to the United States
Source: medRxiv. 2024 Apr 19:2024.04.17.24305856. Preprint. [Version 1] doi: 10.1101/2024.04.17.24305856 (PMC11065033; doi:10.1101/2024.04.17.24305856)
Supplement: Supplementary file 1 [file media-1.pdf]

Variables	Early Cuban Exiles	Freedom Flights	Pre- and Post-Mariel Boatlift	Special Period
N	87	105	106	168
Age	62.49 ± 12.53	57.22 ± 13.88	51.72 ± 16.60	42.45 ± 15.60
Females (N)	48	65	51	83
Males (N)	39	40	55	85
Northeast (N)	3	14	6	5
South (N)	83	90	100	163
West (N)	1	1	0	0

Fig. S1. Descriptive statistics for the Cuban subsample of NLAAS. Here, we report general descriptive statistics for the Cuban subsample of the NLAAS study. Average age and standard deviation was generated while incorporating sampling weights from the NLAAS dataset (see Methods). Note: The individuals included in these analyses are those that met the cutoffs for the migration waves of interest for this study, therefore not all individuals that identify as Cuban in NLAAS are present in these statistics.
