# Supplementary material for: Latiné immigrant heterogeneity: Striking health differences among Cuban refugee/migration waves to the United States
Source: medRxiv. 2024 Apr 19:2024.04.17.24305856. Preprint. [Version 1] doi: 10.1101/2024.04.17.24305856 (PMC11065033; doi:10.1101/2024.04.17.24305856)
Supplement: Supplementary file 2 [file media-2.pdf]

Health Measures	Early Cuban Exiles	Freedom Flights	Pre- and Post-Mariel Boatlift	Special Period
N	87	105	106	168
CARESCORE	1.85 ± 10.08	1.07 ± 6.43	3.47 ± 12.68	0.82 ± 5.76
COGSCORE	1.08 ± 5.86	3.17 ± 12.10	4.67 ± 13.47	1.71 ± 8.58
MOVESCORE	5.61 ± 17.63	4.60 ± 14.73	8.52 ± 20.51	3.63 ± 14.26
OUTROLESCORE	7.96 ± 22.54	10.35 ± 26.88	21.74 ± 39.42	9.45 ± 25.42
SOCIALSCORE	0.64 ± 3.39	1.39 ± 7.05	3.18 ± 12.61	0.81 ± 5.14
DISABILITYSCORE	17.14 ± 45.83	20.58 ± 53.38	41.59 ± 81.76	16.41 ± 44.13
Disabled (N)	23	38	39	37
Not Disabled (N)	64	67	67	131

Fig. S2. Health variable summary statistics for Cuban subsample of NLAAS. This table reports the average, standard deviation, and N (where applicable) for the health measures of interest in this study for the Cuban cohort. These results were generated while incorporating sampling weights from the NLAAS dataset (see Methods). Note: The individuals included in these analyses are those that met the cutoffs for the migration waves of interest for this study, therefore not all individuals that identify as Cuban in NLAAS are present in these statistics. The composite disability score (DISABILITYSCORE) was calculated by adding together all five WHODAS scores for each individual. Individuals were categorized as disabled if they had a composite disability score greater than zero.
