# Supplementary material for: Latiné immigrant heterogeneity: Striking health differences among Cuban refugee/migration waves to the United States
Source: medRxiv. 2024 Apr 19:2024.04.17.24305856. Preprint. [Version 1] doi: 10.1101/2024.04.17.24305856 (PMC11065033; doi:10.1101/2024.04.17.24305856)
Supplement: Supplementary file 3 [file media-3.pdf]

Variables	Early Cuban Exiles	Freedom Flights	Pre- and Post-Mariel Boatlift	Special Period
Medical Discrimination	1.00 ± 0.00	1.34 ± 0.83	1.49 ± 1.12	1.14 ± 0.47
Everyday Discrimination	50.98 ± 5.18	50.69 ± 4.71	50.37 ± 5.74	50.50 ± 5.05

Fig. S3. Discrimination scores summary statistics for the Cuban subsample of NLAAS. This table reports the averages and standard deviations for Medical Discrimination and Everyday Discrimination scores of the Cuban cohort. Averages and standard deviations were generated while incorporating sampling weights from the NLAAS dataset (see Methods). Note: The individuals included in these analyses are those that met the cutoffs for the migration waves of interest for this study, therefore not all individuals that identify as Cuban in NLAAS are present in these statistics.
