# Supplementary material for: Latiné immigrant heterogeneity: Striking health differences among Cuban refugee/migration waves to the United States
Source: medRxiv. 2024 Apr 19:2024.04.17.24305856. Preprint. [Version 1] doi: 10.1101/2024.04.17.24305856 (PMC11065033; doi:10.1101/2024.04.17.24305856)
Supplement: Supplementary file 4 [file media-4.pdf]

**Health Score**	CARESCORE	COGSCORE	MOVESCORE	OUTROLE-SCORE	SOCIALSCORE	DISABILITY-SCORE
CARESCORE						
COGSCORE	r = 0.32<br>p < 2.2e-16					
MOVESCORE	r = 0.56<br>p < 2.2e-16	r = 0.39<br>p < 2.2e-16				
OUTROLESCORE	r = 0.40<br>p < 2.2e-16	r = 0.45<br>p < 2.2e-16	r = 0.59<br>p < 2.2e-16			
SOCIALSCORE	r = 0.43<br>p < 2.2e-16	r = 0.54<br>p < 2.2e-16	r = 0.38<br>p < 2.2e-16	r = 0.37<br>p < 2.2e-16		
DISABILITYSCORE	r = 0.63<br>p < 2.2e-16	r = 0.62<br>p < 2.2e-16	r = 0.81<br>p < 2.2e-16	r = 0.91<br>p < 2.2e-16	r = 0.58<br>p < 2.2e-16	

Table S1. Correlation coefficients for NLAAS health measures analyzed in this study. Here, we report correlation coefficients and p-values for each Pearson's Correlation Test conducted between health measures used within this study. All health measures were significantly positively correlated with one another.
