# Supplementary material for: Latiné immigrant heterogeneity: Striking health differences among Cuban refugee/migration waves to the United States
Source: medRxiv. 2024 Apr 19:2024.04.17.24305856. Preprint. [Version 1] doi: 10.1101/2024.04.17.24305856 (PMC11065033; doi:10.1101/2024.04.17.24305856)
Supplement: Supplementary file 5 [file media-5.pdf]

Dependent Variable (Presence/Absence)	Description	Independent Variable	Control Variables	Odds Ratio	p-value
CARESCORE	An individual's ability to tend to their own personal hygiene	Medical Discrimination (FD21)	Age, Sex, Region	1.89	0.050
COGSCORE	An individual's ability to understand, interact, and cognitively process the world around them	Medical Discrimination (FD21)	Age, Sex, Region	1.85	0.041
MOVESCORE	An individual's ability to move and get around within the home and outside in the wider environment	Medical Discrimination (FD21)	Age, Sex, Region	N/A	0.85
OUTROLESCORE	An individual's ability to complete daily life activities such as work, household activities, and caring for family	Medical Discrimination (FD21)	Age, Sex, Region	N/A	0.33
SOCIALSCORE	An individual's capacity for interpersonal inter-actions	Medical Discrimination (FD21)	Age, Sex, Region	3.87	0.023
DISABILITYSCORE	Composite disability score	Medical Discrimination (FD21)	Age, Sex, Region	N/A	0.94
CARESCORE	An individual's ability to tend to their own personal hygiene	Everyday Discrimination (EDS)	Age, Sex, Region	N/A	0.064
COGSCORE	An individual's ability to understand, interact, and cognitively process the world around them	Everyday Discrimination (EDS)	Age, Sex, Region	N/A	0.069
MOVESCORE	An individual's ability to move and get around within the home and outside in the wider environment	Everyday Discrimination (EDS)	Age, Sex, Region	N/A	0.25
OUTROLESCORE	An individual's ability to complete daily life activities such as work, household activities, and caring for family	Everyday Discrimination (EDS)	Age, Sex, Region	N/A	0.33
SOCIALSCORE	An individual's capacity for interpersonal inter-actions	Everyday Discrimination (EDS)	Age, Sex, Region	N/A	0.073
DISABILITYSCORE	Composite disability score	Everyday Discrimination (EDS)	Age, Sex, Region	N/A	0.091

Table S2. Logistic regression results for discrimination scores. Here we show the results of the logistic regression models for each health measure converted to a binary variable. If a score across any domain was more than zero, those individuals were coded with the presence of a disability. We test if medical discrimination and everyday discrimination scores are associated with the presence of disability, while controlling for age, sex, and region across each model. All models include NLAAS sampling weights.
