# Supplementary material for: Latiné immigrant heterogeneity: Striking health differences among Cuban refugee/migration waves to the United States
Source: medRxiv. 2024 Apr 19:2024.04.17.24305856. Preprint. [Version 1] doi: 10.1101/2024.04.17.24305856 (PMC11065033; doi:10.1101/2024.04.17.24305856)
Supplement: Supplementary file 6 [file media-6.pdf]

Question	Variable	Range	Scale
Frequency treated with less courtesy than others	DS1A	1-6	1: Almost everyday<br>2: At least once a week<br>3: A few times a month<br>4: A few times a year<br>5: Less than once a year<br>6: Never
Frequency treated with less respect than others	DS1B		
Frequency received poorer restaurant service than others	DS1C		
Frequency people act like you are not smart	DS1D		
Frequency people act afraid of you	DS1E		
Frequency people act like you are dishonest	DS1F		
Frequency people act better than you	DS1G		
Frequency called names/insulted	DS1H		
Frequency threatened/harassed	DS1I		

Table S3. Everyday Discrimination Score (EDS) survey questions. Survey questions used to generate the Everyday Discrimination Score (EDS) used as a measure of overall discrimination as part of this study. Participants were the frequency with which they experience discrimination on an everyday basis, with six potential responses: almost everyday, at least once a week, a few times a month, a few times a year, less than once a year, or never. These responses were used to create a numerical value, with a lower value indicating more discrimination and a higher value indicating less discrimination. The scores for survey items DS1A-DS1I were added together to create a composite Everyday Discrimination Score, with a range of 9-54.
