# Supplementary material for: Latiné immigrant heterogeneity: Striking health differences among Cuban refugee/migration waves to the United States
Source: medRxiv. 2024 Apr 19:2024.04.17.24305856. Preprint. [Version 1] doi: 10.1101/2024.04.17.24305856 (PMC11065033; doi:10.1101/2024.04.17.24305856)
Supplement: Supplementary file 7 [file media-7.pdf]

Supplementary Text

Discrimination indices and their limitations

Our study suggests that individuals of the Pre- and Post-Mariel Boatlift may have experienced disproportionately higher discrimination relative to the Early Cuban Exiles, Freedom Flights refugees, and Special Period refugees. Although these results did not reach statistical significance with an alpha of 0.05, how close these results came to being significant merits further investigation.

The survey items that were used to create the Everyday Discrimination Score (EDS) were general questions intended to detect if the individual answering may have been experiencing prejudice or discrimination, but these questions are not specific enough to determine where the discrimination may have been coming from. Discrimination and prejudice operate on many levels. An individual may be discriminated against based on their race, ethnicity, age, gender, sexual orientation, religion, and political leanings (*21*, *30*). These questions do not allow us to understand the context in which these adverse experiences are manifesting thus slightly limiting our ability to understand the specific systems of power that are affecting people. Epidemiological studies excel in their ability to systematically create large, powerful, and comprehensive health datasets to better understand population health on many levels. Adding in qualitative, interview-focused data on a smaller scale can help researchers better contextualize the environment in which health outcomes manifest in regards to its social determinants. The nuance that is captured in these studies allows researchers to understand the complexity of human society as a living, breathing, everyday interactive force.
